# The Prevalence of Mental Health Service Use in Australian Workers with Accepted Workers’ Compensation Claims for Low Back Pain: A Retrospective Cohort Study

**DOI:** 10.1007/s10926-023-10098-3

**Published:** 2023-03-29

**Authors:** Shannon E. Gray, M. Di Donato, L. R. Sheehan, R. Iles, A. Collie

**Affiliations:** Healthy Working Lives Research Group, School of Public Health and Preventive Medicine, Monash University, 553 St Kilda Rd, 3004 Melbourne, Australia

**Keywords:** Low back pain, Workers’ compensation, Injury, Mental health

## Abstract

**Purpose:**

Low back pain (LBP) is a leading cause of disability globally and interferes with work performance and quality of life. For work-related LBP, Australian workers can receive workers’ compensation and access funded healthcare to promote recovery, including mental health services, as there are strong links between chronic LBP and mental health. The objective of this study was to determine the prevalence of funded mental health services for workers with compensated LBP.

**Methods:**

Claims and services data from four Australian workers’ compensation jurisdictions were analysed. Prevalence of accessing at least one mental health service was reported as a percentage of all claims overall and by duration of time loss, age group, sex, financial year of claim lodgement, jurisdiction, socioeconomic status and remoteness. Odds of accessing at least one service was determined using logistic regression.

**Results:**

Almost 10% of LBP claims accessed at least one mental health service (9.7%) with prevalence increasing with time loss. Prevalence was highest in Victoria however a higher percentage of workers with LBP accessed mental health services earlier in Queensland. Odds of accessing services was highest with longest time loss duration, among females and in Queensland. Lower odds were observed in regional areas and among those aged over 56 years.

**Conclusion:**

Findings suggest opportunities for workers’ compensation regulators and insurers to provide greater access to appropriate mental health services alongside physical treatment as standard practice, such as those in more remote locations or earlier in a claim, to improve recovery outcomes for workers with LBP.

**Supplementary Information:**

The online version contains supplementary material available at 10.1007/s10926-023-10098-3.

## Introduction

Low back pain (LBP) is a leading cause of disability, estimated to contribute to 64.9 million years lived with a disability globally in 2017 [1], and interferes with both quality of life and performance at work [2]. Much of the disability burden arising from LBP occurs among working age people [3]. Identifying a cause of LBP can be challenging and most cases are non-specific [4]. While most people recover, it can also be recurrent, with some episodes becoming chronic (i.e., persisting beyond three months) [5].

Musculoskeletal disorders are the most prevalent condition in Australian workers’ compensation schemes, with the back the most commonly affected bodily region [6]. Workers with LBP may be eligible for workers’ compensation if there is a demonstrable link between back pain and employment activities [7].

There are strong links between chronic LBP and mental health. Poor psychological health, presenting as depressed mood, sadness, anger, poor sleep and reduced drive and engagement, may both contribute to LBP and present as a consequence of LBP, particularly when pain symptoms endure [2, 4]. Pre-existing mental health conditions including depression and anxiety can also be exacerbated following physical injury such as LBP [8]. Several factors may contribute to worsening mental health after LBP including ongoing pain, side effects of medication, loss of independence, reduced self-efficacy, engagement in stressful administrative processes such as workers’ compensation, and isolation from friends and colleagues [8–10].

Evidence suggests presence of secondary and pre-existing mental health conditions slows LBP recovery and can delay return to work [10, 11]. Avoidance behaviour, catastrophising and psychological distress in the form of anxiety and depression can exacerbate pain, increasing disability. A biopsychosocial approach, which involves addressing the biophysical (e.g. pain intensity, presence of leg pain), psychological (e.g. distress, attitude) and social components (e.g. social support, workers’ compensation) is particularly important to prevent the worker developing chronic LBP [12] and improve recovery [13].

Secondary mental health conditions following physical injury claims are increasingly recognised as affecting worker recovery by workers’ compensation authorities. Some have sought to minimise the impact of such secondary conditions by identifying workers with psychological support needs, and thus provide tailored support including access to mental health services [14, 15].

Studies using self-report data have shown that up to one in four workers with an occupational musculoskeletal injury report moderate to severe psychological distress [9, 16, 17], and in cases of acute LBP psychological distress is elevated [18]. These same studies report gaps in mental health service use by workers with LBP and psychological distress. A survey of Australian workers’ compensation claimants observed that 20.5% of those with moderate distress and 42.3% with severe distress reported accessing mental health services in the past month [9]. Another study in the state of Victoria, Australia, found that among injured workers with a musculoskeletal compensation claim, around a third experienced severe mental illness, yet only 41.4% of these people accessed mental health services [19].

This prior literature demonstrates a high rate of mental health problems among workers with LBP, and this can delay recovery and affect ability to re-engage in work [11, 20]. These studies also suggest there are gaps in mental health service delivery. To date, studies reporting mental health service use in workers with physical injury compensation claims have been based on small samples, used self-report methods, have not differentiated between different types of musculoskeletal disorders, have not examined determinants of mental health service use, and have not examined whether there are differences in service use between workers’ compensation policy jurisdictions. It is important to ensure adequate support for injured workers with mental health problems to enable planning for suitable service delivery.

This study aims to fill this knowledge gap by using population-based administrative data of those with accepted workers’ compensation claims to describe the prevalence of mental health service use among workers with the most prevalent musculoskeletal disorder of working age, low back pain. The study has multiple aims: to (1) determine the prevalence of accessing compensated mental health services in workers with LBP; (2) describe jurisdictional differences for mental health service access in workers with LBP; and (3) determine the factors associated with accessing mental health services in workers with LBP.

## Methods

### Setting

There are eleven major Australian workers’ compensation schemes. Each state or territory has its own scheme, and there are three for national industries and employers [21]. These fund both wage replacement and ‘reasonable and necessary’ medical and service expenses for workers with an injury or illness attributed to their employment [21]. Mental health services may be funded by compensation schemes for workers with an accepted claim for a mental health condition, or a worker with a musculoskeletal disorder or other physical condition who has developed secondary mental health issues.

Mental health services in Australia can be funded by other sources, including public funding (Medicare, Australia’s national health insurance scheme), private health insurance, or out-of-pocket [22]. Eligibility for funding from each source differs, but importantly, public funding for mental health services is granted on an individual basis by a General Practitioner (i.e., a Primary Care Physician) through a mental health care plan [23]. This option provides individuals with access to up to 10 publicly-funded mental health service treatments per calendar year. Mental health services are therefore accessible to all Australians, but funding is either at the expense of the individual or limited to finite public funding. Publicly-funded mental health services may be subject to waiting periods, however, and therefore accessing services through workers’ compensation could happen sooner.

### Data Source

This study utilises the Monash University Multi-Jurisdictional Workers’ Compensation Database (MJD), which contains de-identified administrative workers’ compensation claim and service payments for musculoskeletal conditions from five Australian workers’ compensation jurisdictions [24]. The database contains claims for LBP, limb fracture and non-specific limb conditions made by workers from Victoria, Queensland, Western Australia, South Australia and Comcare (the national scheme covering federal government employees and some large national employers) that occurred between 1 July 2010 and 30 June 2015. The database also includes details of health services funded by workers’ compensation schemes, which are linked by a de-identified claim linkage key to claim-level data. We have previously described the development of the database including the harmonisation of data across jurisdictions and detailed inclusion and exclusion criteria [24].

### Inclusion Criteria

This study included LBP claims from Victoria, Queensland, South Australia and Western Australia if lodged by the employer between 1 July 2011 and 30 June 2015. These jurisdictions were selected as they maintained detailed service information. Due to 10-day employer excess periods in Victoria and South Australia where workers’ compensation does not cover wage replacement, two weeks of time loss was added to these jurisdictions (as the first two weeks of absence would not be recorded in the dataset) and only claims with at least two weeks’ time loss were included from Queensland and Western Australia. The Australian standard occupational injury and disease coding scheme (i.e., the Type of Occurrence Classification System [25]) was used to identify low back pain claims, as listed in the supplementary table. The primary condition is recorded by case managers based on information provided by physicians upon claim lodgement.

For this study, a mental health service was defined as “an interaction between a mental health professional and a compensated worker”. Services were categorised by provider type and interaction type. Provider types were ‘Psychiatrist’, ‘Psychologist’, ‘Social worker, counsellor or rehabilitation counselling’ (mental health-specific) or ‘Other and unspecified’ (where a service was clearly mental health related, but the provider type could not be identified). Interaction types were ‘Single/one-on-one consultation’, ‘Group consultation or therapy’, or ‘Other interaction type’. Lists of services from each jurisdiction were assessed independently by two reviewers to identify eligible services, based on these definitions, then categorised as above. A third reviewer acted as adjudicator where discrepancies in allocating specific services to service categories occurred. Included services were mental health services associated with included claims, occurring up to 30 days prior or 730 days following claim acceptance. Services were included 30 days prior to claim acceptance to account for any delays from the date of LBP onset and date of claim acceptance. Service eligibility varied between included jurisdictions, with a minimum of two years (730 days) sufficiently common across all jurisdictions.

Duplicate records were excluded, such that services data only included one type of interaction with a particular provider per person per day (e.g., a person could have a one-on-one consultation AND a group consultation with a psychologist on one day, but not two one-on-one psychologist consultations). This accounted for multiple services billed on a single day / invoice. Less than 1% of services were for Other and unspecified providers or Other interaction types, and were therefore not included in analyses.

### Outcome

The outcome of interest was whether a worker had received any workers’ compensation scheme-funded mental health services. A flag was created for each claim if the total count of mental health services was one or more. This flag was used to determine the prevalence (the number of workers receiving at least one mental health service divided by the total number of claims and multiplied by one hundred, expressed as a percentage) and in the logistic regression model.

### Covariates

Covariates were selected based on availability in the dataset and because they have previously been shown to be associated with claim outcomes [26]. Financial year of lodgement was derived from the date of lodgement (e.g., a claim lodged between 1 July 2010 to 30 June 2011 was coded to financial year 2011). Worker sex was categorised as binary (male or female) as this is how it was recorded in the claims dataset. Age at time of lodgement was categorised into the following age groups: 15–25 years, 26–35 years, 36–45 years, 46–55 years, 56–65 years and > 65 years. Jurisdiction represents the workers’ compensation scheme in which the claim was lodged. Worker postcode was mapped to the Socio-Economic Index for Areas to derive the Index of Relative Socioeconomic Advantage and Disadvantage (IRSAD) and grouped into most disadvantaged (lowest quintile), middle three quintiles and most advantaged (highest quintile) [27]. Worker postcode was also linked to the Accessibility/Remoteness Index of Australia to derive remoteness and grouped into major cities of Australia, inner regional Australia, and outer regional Australia/remote/very remote Australia [28].

The duration of time loss (calculated by dividing the number of hours compensated by the number of preinjury work hours per week [26], an appropriate time off work estimate when using administrative data [29]) was also used as a covariate and categorised into the following: 2 to 13 weeks; 13 to 26 weeks; 26 to 52 weeks; 52 to 76 weeks, and; 76 + weeks. These time frames were chosen as they represent typical milestones in the claim process.

### Analysis

Claim information was combined with services (one record-to-many) then collapsed (single record per claim), retaining outcome information. Descriptive characteristics of the claims with at least one mental health service, characteristics of all claims, and the prevalence of accessing at least one mental health service were tabulated. To further present differences by jurisdiction, the total number of claims with corresponding prevalence of at least one mental health service in each jurisdiction by duration of time loss group was also tabulated.

To determine the factors associated with odds of accessing mental health services, logistic regression was performed including all covariates stated above and the mental health service flag as the binary outcome. Results were reported as odds ratios with corresponding 95% confidence intervals.

Missing age, socioeconomic status and remoteness information was imputed using multiple imputation (multivariate imputations by chained equations, five iterations) for the logistic regression model, and statistical significance was set at p < 0.05. All analyses were conducted using R Version 4.0.3 (Vienna, Austria) [30]. Monash University Human Research Ethics Committee approved the project (ID: 17267, November 2018).

## Results

A total of 28,870 claims met the inclusion criteria, of which 2,800 accessed at least one mental health service (prevalence of 9.7%) (Table 1). Fifty-three percent (n = 15,252) had claims lasting less than 13 weeks, while 18% (n = 5,214) lasted at least one year. Almost two-thirds of claims were from males, and claims from those aged 36 to 45 years were most common. Around a third of claims each came from Queensland and Victoria. The majority lived in major cities.

Prevalence of having at least one mental health service increased with time loss duration. Almost two out of five individuals with a claim lasting at least 76 weeks had seen a mental health professional. There was a slight increase in prevalence with increasing year of lodgement. Prevalence varied by jurisdiction from as low as 6.7% in Western Australia to as high as 12.0% in Victoria. Prevalence was similar between socioeconomic statuses but decreased with increased remoteness.

**Table 1:** Description of cohort and prevalence of accessing at least one mental health service

	Number of claims with at least one MH service	Total claims	Prevalence (%)
**Time loss duration group**			
2 to 13 weeks	126	15,252	0.8
13 to 26 weeks	251	4,710	5.3
26 to 52 weeks	544	3,604	15.1
52 to 76 weeks	395	1,446	27.3
76 + weeks	1,484	3,768	39.4
**Financial year of lodgement**			
2012 (i.e., 01 July 2011 to 30 June 2012)	693	7,546	9.2
2013	700	7,511	9.3
2014	728	7,150	10.2
2015	679	6,573	10.3
**Sex**			
Female	982	10,350	9.5
Male	1,818	18,430	9.9
**Age group**			
15–25 years	256	3,514	7.3
26–35 years	720	6,709	10.7
36–45 years	842	7,512	11.2
46–55 years	727	7,250	10.0
56 + years	255	3,794	6.7
**Jurisdiction**			
Queensland	924	9,918	9.3
South Australia	359	3,522	10.2
Victoria	1,117	9,326	12.0
Western Australia	400	6,014	6.7
**Socioeconomic status**			
Most advantaged quintile	399	4,371	9.1
Second to fourth quintiles	1,804	18,642	9.7
Most disadvantaged quintile	472	4,509	10.5
**Remoteness**			
Major cities of Australia	2,027	19,197	10.6
Inner regional Australia	449	5,168	8.7
Outer regional/remote/very remote Australia	198	3,154	6.3
**Total**	**2,800**	**28,780**	**9.7**

MH = mental health

Table 2 shows differences in prevalence by jurisdiction across the different time loss groups. Injured workers in Queensland consistently had the highest prevalence of mental health service use when broken down into duration groups, despite having the third highest overall prevalence. Almost half of the sample from Queensland had accessed at least one service if their claim lasted 52 to 76 weeks, and more than three-quarters if more than 76 weeks. In South Australia the prevalence tripled in claims lasting 52 to 76 weeks compared to 26 to 52 weeks, however this increase was less apparent in Victoria and Western Australia and became more noticeable once a claim exceeded 76 weeks duration.

**Table 2:** Total number of claims and prevalence of having a mental health service within each time loss and jurisdiction group

	2 to 13 weeks	13 to 26 weeks	26 to 52 weeks	52 to 76 weeks	76 + weeks
**Queensland**	6,126, 1.1%	1,900, 8.9%	1,283, 24.7%	361, 48.2%	248, 77.8%
**South Australia**	1,933, 0.5%	477, 4.6%	312, 10.3%	144, 31.9%	656, 38.0%
**Victoria**	3,992, 0.8%	1,517, 2.7%	1,240, 10.2%	472, 20.6%	2,105, 39.0%
**Western Australia**	3,201, 0.4%	816, 2.2%	769, 9.0%	469, 16.6%	759, 29.1%

Increasing time loss duration was associated with higher odds of at least one mental health service (Table 3). Those with claims of at least 76 weeks had 151 times higher odds of having seen a mental health professional than those whose claim lasted 2 to 13 weeks. In the latter two years of claim lodgement, odds of mental health service use were significantly higher. Despite lower prevalence, females had 17% higher odds of accessing mental health services in the adjusted regression model. Compared to those aged 36–45 years, older workers had 46% lower odds of accessing mental health services. Adjusted odds of accessing mental health services was highest in Queensland. There were no significant differences between socioeconomic status. With increasing remoteness there was decreasing odds of accessing mental health services.

**Table 3:** Odds of accessing at least one mental health service

	OR (95% CI)	p-value
**Time loss duration group**		
2 to 13 weeks	Ref	
13 to 26 weeks	6.91 (5.54, 8.66)	< 0.001
26 to 52 weeks	24.48 (20.02, 30.17)	< 0.001
52 to 76 weeks	65.56 (52.57, 82.30)	< 0.001
76 + weeks	151.66 (123.63, 187.64)	< 0.001
**Financial year of lodgement**		
2012	Ref	
2013	1.01 (0.89, 1.15)	0.827
2014	1.17 (1.03, 1.34)	0.015
2015	1.16 (1.02, 1.33)	0.024
**Sex**		
Female	1.17 (1.06, 1.29)	0.002
Male	Ref	
**Age group**		
15–25 years	0.93 (0.78, 1.11)	0.448
26–35 years	1.12 (0.99, 1.27)	0.079
36–45 years	Ref	
46–55 years	0.89 (0.78, 1.00)	0.058
56 + years	0.54 (0.46, 0.64)	< 0.001
**Jurisdiction**		
Queensland	Ref	
South Australia	0.33 (0.28, 0.39)	< 0.001
Victoria	0.30 (0.27, 0.34)	< 0.001
Western Australia	0.21 (0.18, 0.24)	< 0.001
**Socioeconomic status**		
Most advantaged quintile	1.07 (0.94, 1.23)	0.297
Second to fourth quintiles	Ref	
Most disadvantaged quintile	1.00 (0.88, 1.14)	0.959
**Remoteness**		
Major cities of Australia	Ref	
Inner regional Australia	0.71 (0.62, 0.80)	< 0.001
Outer regional/remote/very remote Australia	0.55 (0.46, 0.65)	< 0.001
**Total**		

OR = odds ratio; CI = confidence interval

## Discussion

This study extends our understanding of the prevalence and factors associated with mental health service use by compensated workers with low back pain. Results showed that fewer than 10% of workers with LBP in workers’ compensation systems received compensated mental health services. However, the prevalence was significantly and substantially higher in longer duration claims, with results also highlighting substantial inter-jurisdictional differences. The Australian Institute of Health and Welfare reports that 11% of Australians received Medicare-subsidised mental health-specific services in the 2020/21 financial year, up from 7% to 2010/11 [23], suggesting mental health service use among the general Australian population is increasing much like those with compensated LBP.

Engaging mental health professionals was more likely in Queensland across all durations of time loss. A number of factors could contribute to this difference, including a stronger emphasis on a biopsychosocial approach through case management. Every workers’ compensation jurisdiction in Australia has its own legislation and operational practices [26], therefore it is possible that differences in prevalence and odds of accessing mental health services reflect these. Legislation between the schemes differs, particularly at durations of more than 76 weeks (e.g. the length of time a worker may remain on benefits differs between jurisdictions), thus differences in results between the schemes may be attributable to legislation. There are examples of initiatives in Australian workers’ compensation jurisdictions that address mental health. In South Australia, there is a voluntary mental health support service, targeted at those presenting with mild to moderate anxiety, depression or stress, to support an injured worker to respond “as best as possible” to how their work injury claim and change in circumstances is affecting them [31]. However, this was introduced in the 2018/19 financial year and is therefore not relevant to this study’s cohort. In Queensland, a new initiative (introduced later than the study period) helps injured workers by screening them early in their claim to ascertain whether particular services, including mental health, may help improve recovery [14]. Due to the relative recent introduction of these initiatives, it was unable to be determined whether these impacted prevalence of accessing compensated mental health services using the MJD, and by extension, LBP recovery.

Due to the nature of administrative data, it is unclear whether workers received mental health services in relation to their LBP or secondary psychological conditions. It is possible there are challenges for injured workers with LBP to access mental health services through workers’ compensation as these may not be seen as directly related to the primary injury. Whilst it has been known for some time that psychological distress is important to consider in LBP aetiology, and can become the more significant issue for treatment and management [10, 32], only 9.7% of all claims accessed compensated mental health services. Rather than psychological distress, however, some workers with LBP may be seeing psychologists who specialise in pain management. Best practice guidelines recommend applying a biopsychosocial approach to the treatment of LBP to prevent poor outcomes and development of persistent LBP, however a mental health professional is not necessarily the provider considering the psychosocial components [13, 15, 33, 34]. Due to the recommended approach to consider the psychological health of an injured individual to improve recovery and that only one in ten of these claims for LBP receives compensated mental health care, there is an opportunity for more workers with LBP to receive and benefit from mental health services. Further, recovery is likely to be accelerated with improved function and return to work outcomes by providing services earlier in the claim.

Year of claim lodgement, age, sex, remoteness, and duration of claim were also significantly associated with accessing compensated mental health services. Mental health services were more commonly delivered to those living closer to major cities, which could reflect availability of specialist mental health services [23]. Whilst prevalence was similar, females had significantly higher odds of having accessed a compensated mental health service after adjustment for other covariates. Previous studies have found females experience higher distress following injury [17] and are more likely to access mental health services [9]. As the year of lodgement became more recent, accessing mental health services became more prevalent. It is possible that through time there has been increased awareness of the importance of addressing mental health issues to improve worker outcomes, particularly for chronic conditions. Alternatively, this increase could be driven by policy or practice changes within workers’ compensation jurisdictions. For example, it is possible that once workers have reached a particular claim duration their case managers might recommend seeing a mental health professional.

This is the first known study to report the prevalence of accessing mental health services via workers’ compensation with a cohort of workers with compensated LBP. In order to obtain the findings, an Australian-first large-scale administrative dataset of multiple workers’ compensation jurisdictions’ claims and services data was created. Further, robust statistical techniques were used to report outcomes.

A limitation of the study is that we were only able to capture specialist mental health services and not instances where a mental health plan had been developed with a General Practitioner (and no referral to specialist services occurred). Any psychological services that were funded outside the workers’ compensation system were also unable to be captured. Therefore, due to these factors prevalence of accessing mental health help is likely to be higher. Further, we have no clinical information in the data and therefore do not know the reasons why the injured worker consulted with the mental health professional. However, as these services were funded by workers’ compensation they are likely treating secondary mental health issues related to the LBP and work disability. Occupation and industry were not well coded in all jurisdictions and could therefore not be added as covariates, despite potentially explaining some of the differences. Finally, the mental health services reported in this study are limited to the accuracy of those recording the initial information.

The next step is to determine whether consulting with mental health professionals throughout the recovery period in this cohort has a relationship with return to work and health outcomes, as evidenced elsewhere [35]. Findings do suggest, however, that there is a clear opportunity to support not only the physical recovery of an injured worker, but also support the psychological challenges that experiencing occupational injury may present. Determining the impact of concurrent mental health care could demonstrate the value to workers’ compensation insurers and regulators in offering psychological support during recovery.

## Conclusion

This study utilised health service use data from four Australian workers’ compensation jurisdictions to show that fewer than 10% of injured workers with workers’ compensation claims for LBP accessed compensated mental health services throughout the two years since their claim was accepted. This is despite numerous studies showing that up to 40% of workers with occupational musculoskeletal injury experience moderate to severe psychological distress, which is more elevated among those with acute LBP [9, 16–18]. Access to compensated mental health services varied between jurisdictions. The factors identified in this study of those more or less likely to access mental health services via workers’ compensation may be helpful to direct future services to improve recovery, such as to those in more remote locations. Results suggest opportunities for workers’ compensation regulators and insurers to provide greater access to appropriate mental health care alongside physical treatment as standard practice, earlier in a claim.

## Electronic Supplementary Material

Below is the link to the electronic supplementary material.

Supplementary Material 1

## References

[1] Wu A, March L, Zheng X (2020). Global low back pain prevalence and years lived with disability from 1990 to 2017: estimates from the global burden of Disease Study 2017. Ann Transl Med.

[2] Ehrlich GE (2003). Low back pain. Bull World Health Organ.

[3] Australian Institute of Health and Welfare, Australian Burden of Disease Study: impact and causes of illness and death in Australia 2018. 2021, Australian Burden of Disease Study series no. 23. Cat. no. BOD 29: Canberra: AIHW.

[4] Hartvigsen J, Hancock MJ, Kongsted A (2018). What low back pain is and why we need to pay attention. Lancet.

[5] Australian Commission on Safety and Quality in Health Care (2022). Low back Pain Clinical Care Standard.

[6] Safe Work Australia (2019). Australian workers’ compensation Statistics 2018-19.

[7] Oakman J, Clune S, Stuckey R (2019). Work-related musculoskeletal disorders in Australia, 2019.

[8] Gu JK, Charles LE, Fekedulegn D (2020). Occupational injury and psychological distress among U.S. workers: the National Health interview Survey, 2004–2016. J Saf Res.

[9] Collie A, Sheehan L, Lane TJ (2020). Psychological distress in workers’ compensation claimants: prevalence, predictors and mental health service use. J Occup Rehabil.

[10] Hope P, Forshaw MJ (1999). Assessment of psychological distress is important in patients presenting with low back pain. Physiotherapy.

[11] Besen E, Young AE, Shaw WS (2015). Returning to work following low back pain: towards a model of individual psychosocial factors. J Occup Rehabil.

[12] Chou R, Shekelle P (2010). Will this patient develop persistent disabling low back pain?. JAMA.

[13] Oliveira CB, Maher CG, Pinto RZ (2018). Clinical practice guidelines for the management of non-specific low back pain in primary care: an updated overview. Eur Spine J.

[14] Work Cover Queensland. Recovery Blueprint. Available from: https://www.worksafe.qld.gov.au/about/who-we-are/workcover-queensland/workcover-queensland-research-initiatives/recovery-blueprint. Accessed [cited May 11 2022].

[15] Thumula V, Negrusa S (2022). A primer on behavioral Health Care in Workers’ compensation.

[16] Leijon O, Mulder M (2009). Prevalence of low back pain and concurrent psychological distress over a 16-year period. Occup Environ Med.

[17] Richardson AE, Derrett S, Samaranayaka A (2021). Prevalence and predictors of psychological distress following injury: findings from a prospective cohort study. Inj Epidemiol.

[18] Shaw WS, Hartvigsen J, Woiszwillo MJ (2016). Psychological distress in acute low back pain: a review of measurement scales and levels of distress reported in the first 2 months after pain onset. Arch Phys Med Rehabil.

[19] Orchard C, Carnide N, Mustard C (2020). Prevalence of serious mental illness and mental health service use after a workplace injury: a longitudinal study of workers’ compensation claimants in Victoria, Australia. Occup Environ Med.

[20] Franche RL, Carnide N, Hogg-Johnson S (2009). Course, diagnosis, and treatment of depressive symptomatology in workers following a workplace injury: a prospective cohort study. Can J Psychiatry.

[21] Safe W, Australia (2018). Comparison of workers’ compensation arrangements in Australia and New Zealand 2017.

[22] Bohm K, Schmid A, Gotze R (2013). Five types of OECD healthcare systems: empirical results of a deductive classification. Health Policy.

[23] Australian Institute of Health and Welfare. Mental health services in Australia. Available from: https://www.aihw.gov.au/reports/mental-health-services/mental-health-services-in-australia/report-contents/medicare-subsidised-mental-health-specific-services. Accessed [cited 29 August 2022].

[24] Di Donato M, Iles R, Buchbinder R (2021). Prevalence, predictors and wage replacement duration associated with diagnostic imaging in australian workers with accepted claims for low back pain: a retrospective cohort study. J Occup Rehabil.

[25] Australian Safety and Compensation Council. Type of occurrence classification system 3rd Edition, revision 1. Canberra; 2008.

[26] Collie A, Lane TJ, Hassani-Mahmooei B (2016). Does time off work after injury vary by jurisdiction? A comparative study of eight australian workers’ compensation systems. BMJ Open.

[27] Australian Bureau of Statistics. Postal area, indexes, SEIFA (socio-Economic indexes for Areas) 2011, in Census of Population and Houseing: Socio-Economic indexes for areas (SEIFA), Australia, 2011. Canberra; 2013.

[28] Australian Bureau of Statistics. 1270.0.55.006 - Australian Statistical Geography Standard (ASGS): Correspondences, July 2011. Available from: http://www.abs.gov.au/AUSSTATS/abs@.nsf/DetailsPage/1270.0.55.006July%202011?OpenDocument. Accessed [cited October 9 2022].

[29] Krause N, Dasinger LK, Deegan LJ (1999). Alternative approaches for measuring duration of work disability after low back injury based on administrative workers’ compensation data. Am J Ind Med.

[30] Core Team R. R: A Language and Environment for Statistical Computing. R Foundation for Statistical Computing: Vienna, Austria; 2020.

[31] ReturnToWorkSA. Supporting your mental wellbeing: Fast access to support services. Available from: https://www.rtwsa.com/__data/assets/pdf_file/0008/109169/Low-intensity-mental-health-DL-Dec-19-WEB.pdf. Accessed [cited October 9 2022].

[32] Hope P (2002). Assessment and treatment of patients presenting with low back pain and accompanying psychological distress: an evidence-based approach. Physiotherapy.

[33] National Institute for Health and Care Excellence (NICE). Low back pain and sciatica in over 16s: assessment and management. Available from: https://www.nice.org.uk/guidance/ng59/resources/low-back-pain-and-sciatica-in-over-16s-assessment-and-management-pdf-1837521693637. Accessed [cited 2022 May 11]. 33090750

[34] NSW Agency for Clinical Innovation (2016). Management of people with acute low back pain: model of care.

[35] Brown TT, Ahn C, Huang H (2020). Reducing the prevalence of low-back pain by reducing the prevalence of psychological distress: evidence from a natural experiment and implications for health care providers. Health Serv Res.

