# Supplementary material for: The Prevalence of Mental Health Service Use in Australian Workers with Accepted Workers’ Compensation Claims for Low Back Pain: A Retrospective Cohort Study
Source: J Occup Rehabil. 2023 Mar 29;33(3):602–9. doi: 10.1007/s10926-023-10098-3 (PMC10495495; doi:10.1007/s10926-023-10098-3)
Supplement: Supplementary file 1 — Supplementary Material 1 [file 10926_2023_10098_MOESM1_ESM.docx]

**Supplementary table: Criteria for selection of low back pain cases**

| Nature of Injury | 228 – Trauma to muscles and tendons, NEC OR,  229 – Trauma to muscles and tendons, US OR,  239 – Soft tissue injuries due to trauma or unknown mechanisms with insufficient information to code OR,  422 – Disc displacement, prolapse, degeneration or hernia OR,  459 – Back pain, lumbago, and sciatica OR,  533 – Muscle / tendon strain (non-traumatic) |
| --- | --- |
| Location of Injury | 311 – Lower back |
| Mechanism of Injury | Any |
| Agency of Injury | Any |

*NEC = not elsewhere classified; US = unspecified
